# Key players of singlet oxygen-induced cell death in plants

**DOI:** 10.3389/fpls.2015.00039

**Published:** 2015-02-04

**Authors:** Christophe Laloi, Michel Havaux

**Affiliations:** ^1^Laboratoire de Génétique et Biophysique des Plantes, Institut de Biologie Environnementale et Biotechnologie, Commissariat à l’Énergie Atomique et aux Énergies AlternativesMarseille, France; ^2^CNRS, UMR 7265 Biologie Végétale et Microbiologie EnvironnementalesMarseille, France; ^3^Aix Marseille UniversitéMarseille, France; ^4^Laboratoire d’Ecophysiologie Moléculaire des Plantes, Institut de Biologie Environnementale et Biotechnologie, Commissariat à l’Énergie Atomique et aux Énergies AlternativesSaint-Paul-lez-Durance, France

**Keywords:** singlet oxygen, oxidative stress, cell death, acclimation, EXECUTER, β-cyclocitral, phytohormones, oxylipins

## Abstract

The production of reactive oxygen species (ROS) is an unavoidable consequence of oxygenic photosynthesis. Singlet oxygen (^1^O_2_) is a highly reactive species to which has been attributed a major destructive role during the execution of ROS-induced cell death in photosynthetic tissues exposed to excess light. The study of the specific biological activity of ^1^O_2_ in plants has been hindered by its high reactivity and short lifetime, the concurrent production of other ROS under photooxidative stress, and limited *in vivo* detection methods. However, during the last 15 years, the isolation and characterization of two ^1^O_2_-overproducing mutants in *Arabidopsis thaliana*, *flu* and *ch1*, has allowed the identification of genetically controlled ^1^O_2_ cell death pathways and a ^1^O_2_ acclimation pathway that are triggered at sub-cytotoxic concentrations of ^1^O_2_. The study of *flu* has revealed the control of cell death by the plastid proteins EXECUTER (EX)1 and EX2. In *ch1*, oxidized derivatives of β-carotene, such as β-cyclocitral and dihydroactinidiolide, have been identified as important upstream messengers in the ^1^O_2_ signaling pathway that leads to stress acclimation. In both the *flu* and *ch1* mutants, phytohormones act as important promoters or inhibitors of cell death. In particular, jasmonate has emerged as a key player in the decision between acclimation and cell death in response to ^1^O_2_. Although the *flu* and *ch1* mutants show many similarities, especially regarding their gene expression profiles, key differences, such as EXECUTER-independent cell death in *ch1*, have also been observed and will need further investigation to be fully understood.

## INTRODUCTION

Singlet oxygen (^1^O_2_) is an unavoidable byproduct of oxygenic photosynthesis. This reactive oxygen species (ROS) is produced in energy transfer reactions from the excited triplet state of chlorophyll molecules or their precursors to molecular oxygen. ^1^O_2_ is highly reactive and engages readily with a variety of biomolecules, especially those containing double bonds (Triantaphylidès and Havaux, 2009), and results in reduced photosynthetic efficiency and ultimately cell death. ^1^O_2_ is believed to be the main ROS produced in the chloroplasts under stress and excess light, playing a major destructive role during the execution of ROS-induced cell death in leaf tissues (Triantaphylidès et al., 2008). Plants have developed various scavenging systems to protect themselves against the toxic effects of ^1^O_2_. Carotenoids, tocopherols and plastoquinones which are present in the thylakoid membranes are thought to play an essential role in quenching ^1^O_2_ (Krieger-Liszkay et al., 2008; Triantaphylidès and Havaux, 2009). Other scavengers such as ubiquinol, ascorbate, and glutathione may also quench ^1^O_2_.

While the intracellular signaling functions of the long-lived ROS hydrogen peroxide (H_2_O_2_) and its implication in the regulation of cell death have been established for some time (Levine et al., 1994; Thordal-Christensen et al., 1997), a similar function for ^1^O_2_ has only been recognized more recently in plants (op den Camp et al., 2003; Wagner et al., 2004). This delayed knowledge stems from the fact that the study of the specific biological activity of ^1^O_2_ in plants is hampered, firstly, by the high reactivity and short lifetime of this ROS [∼4 μs in water (Wilkinson et al., 1995), likely lower than 0.5–1 μs in plant cells (Bisby et al., 1999; Redmond and Kochevar, 2006; Li et al., 2012; Telfer, 2014)], and, secondly, by the concurrent production of other ROS under photooxidative stress. Nevertheless, our comprehension of the cellular responses to ^1^O_2_ in plants has dramatically progressed during the last decade, thanks to the use of *Arabidopsis thaliana* (hereafter, *Arabidopsis*) mutants characterized by the conditional accumulation of ^1^O_2_ within the chloroplast. This mutant approach has revealed that, besides its direct toxicity, ^1^O_2_ can also function as a signal molecule (Kim et al., 2008). Depending on the levels of ^1^O_2_ production induced by light in these mutants, the ^1^O_2_-triggered signaling pathway was found to lead to different cell death responses or to an acclimation process, with phytohormones appearing to be major players in the orientation of the ^1^O_2_ signaling pathway toward a particular response. The present review summarizes the main findings on these ^1^O_2_-induced cell death and acclimation mechanisms and their control by phytohormones.

## GENETICALLY CONTROLLED ^1^O_2_-TRIGGERED CELL DEATH IN *Arabidopsis*: LEARNING FROM THE *flu* MUTANT

**FIGURE 1:**
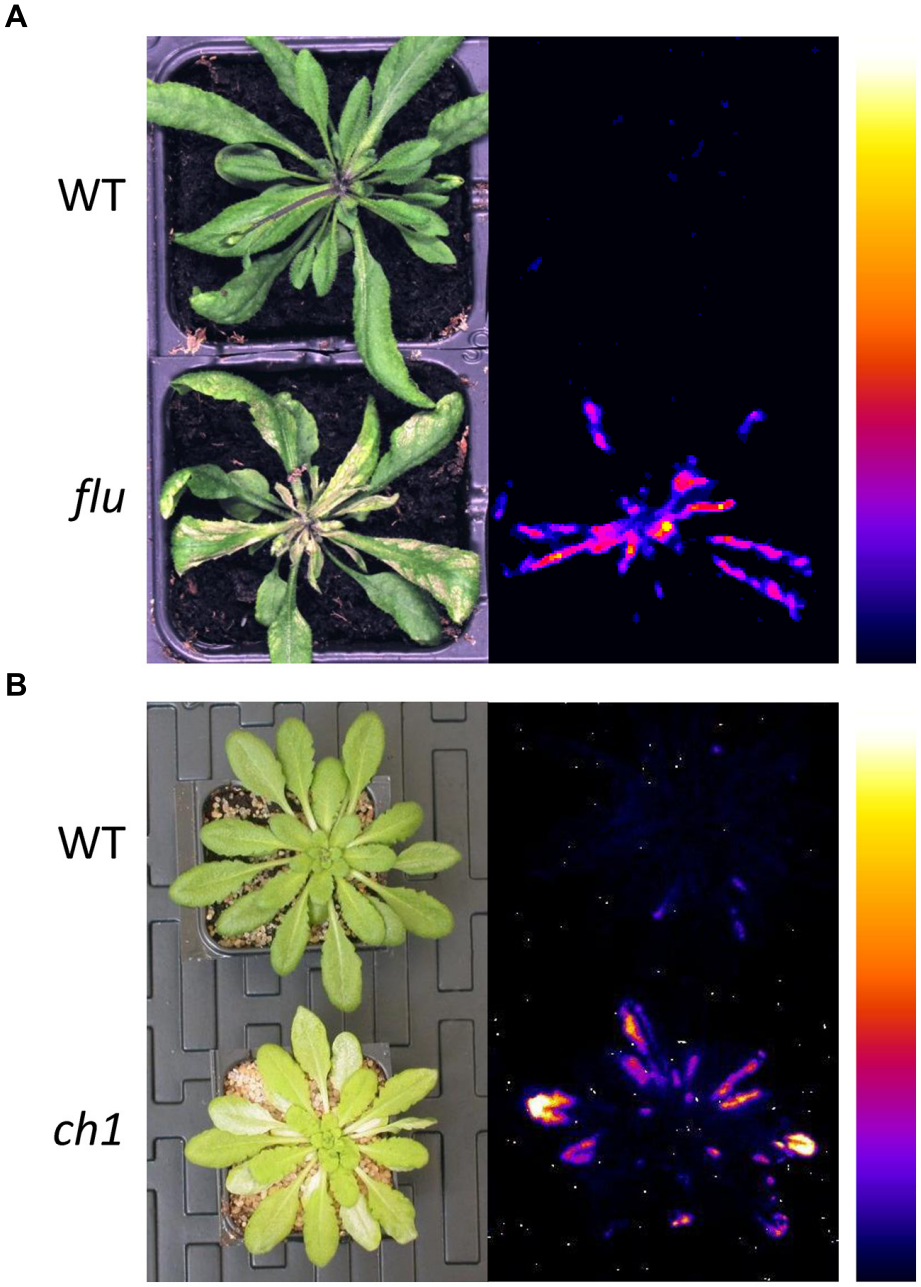
**Photosensitivity of the ^1^O_2_-overproducing *Arabidopsis* mutants, *flu* and *ch1*. (A)** Wild-type (WT) and *flu* mutant plants after an 8 h dark/light transition, showing leaf damage (2 days after the dark-to-light shift, left) and lipid peroxidation as measured by autoluminescence imaging (2 h after the dark-to-light shift, right). **(B)** WT and *ch1* mutant plants after high light stress (1000 μmol photons m^-2^ s^-1^ for 2 days), showing leaf bleaching (left) and lipid peroxidative damage (right). No luminescence and no leaf damages were detected in WT and the mutant plants before the light treatments. The color palette on the right indicates the intensity of the luminescence signal from low (dark blue) to high (white). The signal intensity is indicative of the amount of lipid peroxides present in the sample (Birtic et al., 2011). Adapted from Ramel et al. (2013a) (Copyright American Society of Plant Biologists, http://www.plantcell.org) and completed.

A major breakthrough in understanding the intracellular signaling function of ^1^O_2_ was the isolation and characterization of the conditional *fluorescent* (*flu*) mutant of *Arabidopsis*, which is defective in the nuclear-encoded FLU protein involved in the negative feedback control of the Mg^2+^ branch of tetrapyrrole biosynthesis (Meskauskiene et al., 2001; Meskauskiene and Apel, 2002). In contrast to wild-type plants, *flu* plants are no longer able to restrict the synthesis of protochlorophyllide (Pchlide) in the dark. As a consequence, free Pchlide accumulates in the dark and then acts as a photosensitizer upon re-illumination, generating ^1^O_2_ in a rapid (and probably transient) manner within the chloroplasts (**Figure 1**). The *flu* mutant has two other important properties. First, *flu* plants under continuous light, where Pchlide is immediately photo-reduced to chlorophyllide and hence does not accumulate, have a wild-type phenotype. Second, the amount of ^1^O_2_ generated is proportional to the amount of Pchlide and hence to the extent of the dark period. Therefore, the conditional *flu* mutant is a powerful tool for the generation of different quantities of ^1^O_2_ in photosynthetic tissues in a noninvasive and controlled manner. This makes it possible to study the stress response triggered by the release of a specific quantity of ^1^O_2_ inside plastids simply by growing the plants under continuous light till they reach the developmental stage of interest, transferring them to the dark for a defined time period, and then re-illuminating them.

In this way, the release of ^1^O_2_ during re-illumination of *flu* plants grown under continuous light and transferred to the dark for 8 h was initially shown to result in a rapid induction of specific sets of nuclear genes that were not activated by other treatments generating different ROS inside plastids (op den Camp et al., 2003; Gadjev et al., 2006; Laloi et al., 2006). In addition, two obvious stress responses were observed in *flu* plants following the release of ^1^O_2_: a transient inhibition of growth almost immediately after the beginning of re-illumination, and a cell death response. Quite unexpectedly, the growth arrest and cell death responses in the *flu* mutant were found to result from a genetic program that involves the EXECUTER proteins (EX1 and EX2), rather than from the direct toxicity of ^1^O_2_ (Wagner et al., 2004; Lee et al., 2007). The EXECUTER pathway also promoted cell death in wild-type plants treated with DCMU (3-(3,4-dichlorophenyl)-1,1-dimethylurea) (Wagner et al., 2004), a herbicide that blocks photosynthetic electron transport at the level of the photosystem II (PSII) primary quinone electron acceptor, thus leading to the increased production of ^1^O_2_ in PSII reaction centers (Fufezan et al., 2002). Cytological changes that follow the onset of ^1^O_2_ production have been recently shown to include the sequential rapid loss of chloroplast integrity that precedes the rupture of the central vacuole and the final collapse of the cell (Kim et al., 2012). Inactivation of the two plastid proteins EXECUTER in the *flu* mutant abrogates these responses, indicating that disintegration of chloroplasts is due to EX-dependent signaling rather than ^1^O_2_ directly (Kim et al., 2012). Interestingly, it was shown that the initial light-dependent release of ^1^O_2_ was not sufficient to trigger the onset of cell death, but had to act together with a second concurrent blue light reaction that requires the activation of the UVA/blue light- absorbing photoreceptor CRY1 (Danon et al., 2006).

## CELL DEATH-PROMOTING AND -INHIBITORY SIGNALS IN *flu*

**FIGURE 2:**
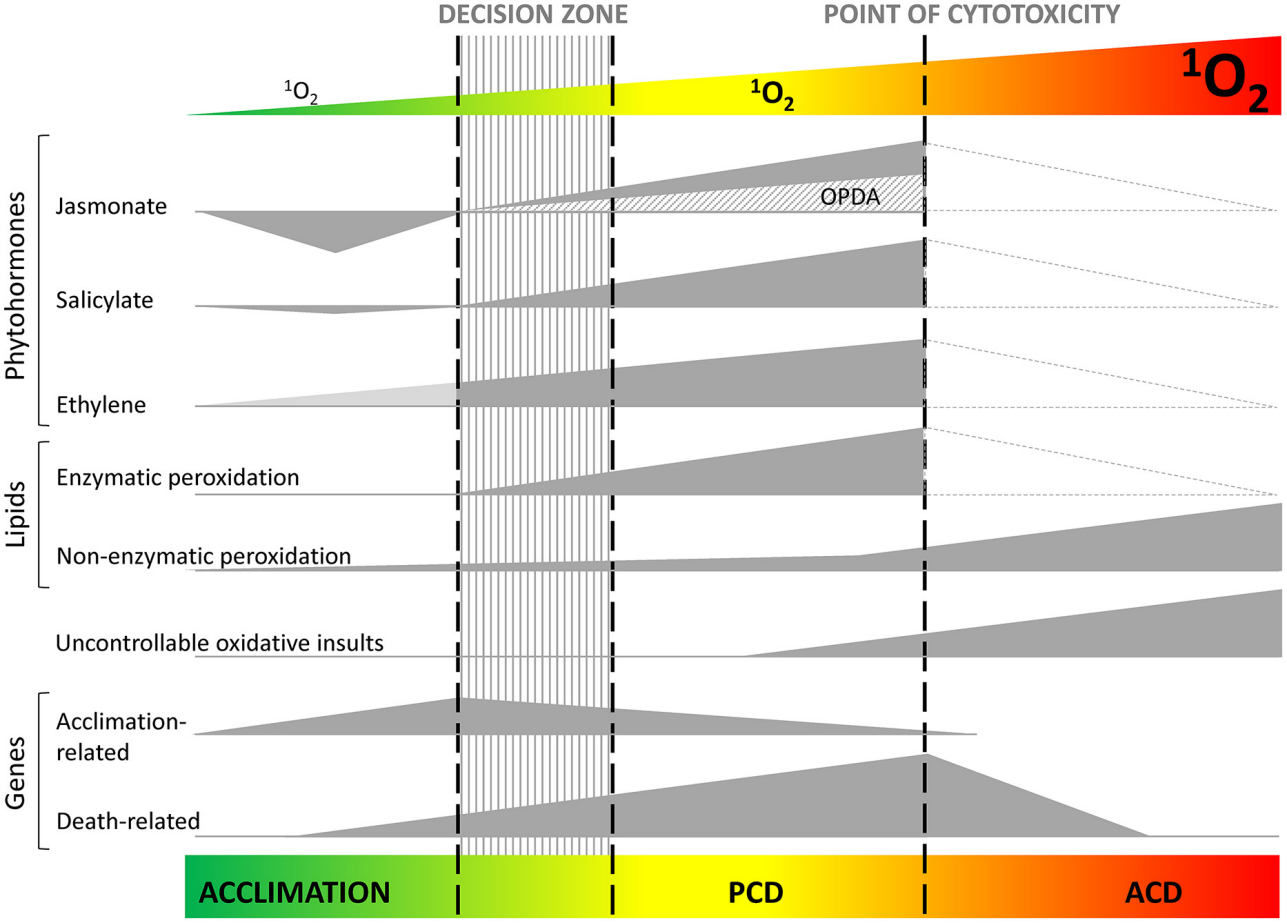
**A model for the cellular responses to different ^**1**^O_**2**_ levels.** Depending on the level of ^1^O_2_, the plant cell might trigger an acclimation response or a programmed cell death (PCD) program, or, in case of extreme ^1^O_2_ production, will succumb in a completely uncontrollable manner to the so-called ‘accidental cell death’ (ACD), due to a general oxidation that leads to the loss of structural integrity. PCD and acclimation, which are both initiated by genetically encoded machinery, are controlled by the phytohormones jasmonic acid (JA), salicylic acid (SA), and ethylene (ET). JA, SA, and ET seem to work as PCD-promoting signals, while the JA precursor OPDA (and dnOPDA) might counteract the JA-effect and work as inhibitory signals. JA seems to play a central role in the decision between acclimation and PCD. Evidence suggests that ^1^O_2_ production sites, production rates and production times will influence this model. The dotted lines in the ACD zone indicate predicted changes in phytohormone concentrations which still need to be substantiated by experimental data.

Several genes that are up-regulated after the release of ^1^O_2_ in the *flu* mutant encode proteins involved in the biosynthesis or signaling of the phytohormones ethylene (ET), salicylic acid (SA), and jasmonic acid (JA), suggesting that these phytohormones could be involved in triggering cell death. This hypothesis is further supported by the increased concentrations of oxylipins [12-oxo-phytodienoic acid (OPDA), dinor-OPDA (dnOPDA), and JA] in *flu* soon after re-illumination. The oxylipin increase was followed by a slower increase in SA, with variations depending on the developmental stage (Danon et al., 2005; Ochsenbein et al., 2006; **Figure 2**). The possible involvement of these phytohormones in regulating cell death was tested by combining genetic and pharmacological approaches (Danon et al., 2005). *flu* mutant seedlings expressing the *NahG* transgene, which encodes a salicylate hydroxylase that converts SA to catechol, were partially protected from the death provoked by the release of ^1^O_2_, suggesting a requirement for SA in this process. However, a role for the possibly increased levels of catechol in reducing the susceptibility was not completely excluded (van Wees and Glazebrook, 2003; Danon et al., 2005). A similar partial protection from cell death was found when ET biosynthesis was blocked in the *flu* mutant by the addition of L-aminoethoxyvinylglycine (AVG), a potent inhibitor of 1-Aminocyclopropane-1-carboxylic acid (ACC) synthase. These treatments were additive because *flu NahG* plants grown on a medium containing AVG were almost fully protected from cell death. This suggests that there is a synergistic interaction between the SA and ET signaling pathways during the regulation of cell death induced by ^1^O_2_ in *flu* (Danon et al., 2005).

The possible role of oxylipins in the ^1^O_2_ cell death was investigated by crossing *flu* with the JA-depleted mutant *opr3* and with the JA-, OPDA-, and dnOPDA-depleted *dde2-2* mutant defective in the *ALLENE OXIDE SYNTHASE* gene. The analysis of cell death in the double mutant lines, in combination with or without the addition of exogenous JA, revealed that, in contrast to the JA-induced suppression of superoxide/H_2_O_2_-dependent cell death, JA promotes the ^1^O_2_-mediated cell death reaction. Other oxylipins, most likely OPDA/dnOPDA, were found to antagonize the death-promoting activity of JA (Danon et al., 2005; **Figure 2**).

Other ROS seem to have an inhibitory effect on ^1^O_2_-triggered cell death, as revealed in *flu* plants overexpressing the thylakoid-bound ascorbate peroxidase (tAPX), an H_2_O_2_-scavenging enzyme (Murgia et al., 2004). In *flu* overexpressing tAPX, the intensity of ^1^O_2_-mediated cell death was increased when compared with the *flu* line, suggesting that H_2_O_2_ either directly or indirectly suppresses ^1^O_2_-triggered cell death (Laloi et al., 2007). The inhibitory effect of H_2_O_2_ may explain why, in wild-type plants exposed to photooxidative stress conditions that trigger the simultaneous production of ^1^O_2_ and H_2_O_2,_ cell death is delayed by several days from the onset of the light stress. In contrast, the generation of ^1^O_2_ in *flu* occurs without a concomitant production of H_2_O_2_ and is rapidly followed by cell death (Kim et al., 2012). In the microalga *Chlamydomonas reinhardtii*, H_2_O_2_ production induced by high light treatment was shown to promote carotenoid synthesis, thus enhancing ^1^O_2_ scavenging ability and hence delaying photooxidative damage and cell death (Chang et al., 2013). This delay appears to be important for allowing the plant enough time to activate adaptive responses before initiating a cell death program if cellular homeostasis cannot be restored. The antagonistic effect of H_2_O_2_ on ^1^O_2_-triggered cell death might thus contribute to the overall robustness of wild-type plants exposed to photooxidative stress conditions. Therefore, the relative amount of different ROS, rather than the concentration of one particular ROS, seems to determine the activation of either a stress defense or cell death programs. This hypothesis is developed in greater detail in the context of leaf senescence by Sabater and Martín (2013) in this Research Topic.

## ^1^O_2_-TRIGGERED ACCIDENTAL CELL DEATH IN *flu*

The *flu* mutant was isolated and first characterized during a genetic screen for etiolated mutant seedlings that are no longer able to restrict the accumulation of Pchlide in the dark and hence emit strong red Pchlide fluorescence when exposed to blue light. In etiolated seedlings, introduction of the *ex1* mutation does not suppress seedling lethality of the *flu* mutation. The ineffectiveness of the *ex1* mutation under these conditions has been attributed to the fact that *flu* and *flu ex1* etiolated seedlings accumulate about four times more Pchlide than seedlings grown in continuous light and transferred to the dark for 8 h, which are the initial light conditions under which the *ex1* mutation was shown to suppress cell death and growth inhibition (Wagner et al., 2004; Przybyla et al., 2008). Therefore, *flu* plants exposed to an 8 h dark period then re-illuminated appear to produce a relatively low level of ^1^O_2_ which has limited cytotoxic effects, but which is nevertheless able to trigger a genetically controlled cell death program that is regulated by the EXECUTER proteins. Conversely, etiolated *flu* seedlings transferred to light generate a high level of ^1^O_2_ that is cytotoxic and causes irreversible damage to cellular components (Przybyla et al., 2008). Thus, at least two types of cell death can be triggered by ^1^O_2_ in *flu*: cells exposed to very high levels of ^1^O_2_ succumb in a EXECUTER-independent and uncontrollable manner, a process generally referred to as *“*accidental cell death” (ACD; van Doorn et al., 2011; Galluzzi et al., 2015); alternatively, in cells exposed to a lower production of ^1^O_2_ inside plastids, a programmed cell death (PCD) is initiated that involves the genetic control by EXECUTER proteins. Both types of cell death may coexist at the level of a tissue, organ or the whole plant, significantly complicating the identification of reliable molecular markers for cell death type.

Measurements of oxylipins, the oxidation products of polyunsaturated fatty acids (PUFAs), further support the concept of two types of ^1^O_2_-triggered cell death. Etiolated *flu* seedlings transferred to light accumulate large amounts of non-enzymatically formed oxylipins, the hydroxyoctadecadienoic acids (HODE) 10-HODE and 12-HODE and the hydroxyoctadecatrienoic acids 10-HOTE and 15-HOTE. This indicates that under these extreme conditions the toxic effects of ^1^O_2_ prevail (Przybyla et al., 2008). Interestingly, 10-HOTE and 15-HOTE, which are specific products of ^1^O_2_-mediated PUFA oxidation, also accumulate in wild-type plants exposed to simultaneous high light and cold conditions (Havaux et al., 2009). This suggests that ^1^O_2_ is the major ROS responsible for lipid peroxidation under these conditions (Triantaphylidès et al., 2008). In contrast to etiolated *flu* seedling, *flu* plants kept in the dark for 8 h then re-illuminated accumulate enzymatically produced oxylipins, such as 13-HOTE, 13-HODE, OPDA, and JA; the products of non-enzymatic lipid peroxidation are not detected (Przybyla et al., 2008).

The discovery of at least two different types of ^1^O_2_-triggered cell death in the *flu* mutant raises the question of whether ^1^O_2_ levels lower than those that trigger genetically controlled cell death could activate an acclimation response. The characterization of another ^1^O_2_–producing mutant, *ch1*, has allowed us to answer this question.

## THE *Arabidopsis ch1* MUTANT, AN ALTERNATIVE MODEL TO *flu*

Impairment of the *CAO* gene, encoding chlorophyll *a* oxygenase, in the *Arabidopsis chlorina1* (*ch1*) mutant results in the absence of chlorophyll *b* and in a pale-green phenotype (Espineda et al., 1999). Because functional PSII antennae cannot assemble in the absence of chlorophyll *b* (Plumley and Schmidt, 1987), the *ch1* mutant is completely deficient in functional light-harvesting antenna complex of PSII (LHCII; Havaux et al., 2007). The only PSII antenna protein detected in significant amounts in *ch1* is Lhcb5, but this protein is not assembled with pigments and is therefore not functional in this mutant (Havaux et al., 2007; **Figure 1**). Thus, PSII is composed solely of its reaction center core (with the internal antennae) in the leaves of *ch1*. These alterations have relatively little effect on photosynthetic electron flow at high photon flux densities (PFDs; Havaux et al., 2007; Kim et al., 2009; Ramel et al., 2013a). Loss of the normal structural architecture around the PSII reaction centers in chlorophyll b–less plant mutants has significant effects on the functionality of the PSII complexes, including loss of non-photochemical energy quenching (NPQ), impaired oxidizing side of PSII, and reduced grana stacking (Havaux and Tardy, 1997; Kim et al., 2009). LHCI antennae are less dependent on chlorophyll *b* for assembly than LHCIIs and, consequently, the PSI units retain part of their LHCIs in *ch1* mutant leaves (Havaux et al., 2007; Takabayashi et al., 2011).

The *ch1* mutant was found to be highly photosensitive, exhibiting leaf damage and cell death under light conditions (1000 μmol photons m^-2^ s^-1^ for 2 days) that had very limited effects on wild-type leaves (Havaux et al., 2007; **Figure 1**). This effect is attributable to an increased release of ^1^O_2_ from the ‘naked’ PSII centers in the absence of the protective mechanisms associated with the light-harvesting system, such as non-photochemical energy quenching (NPQ). Accordingly, inhibition of NPQ in the *Arabidopsis npq4* mutant has been reported to increase the oxidation of β-carotene by ^1^O_2_ in the PSII reaction centers (Ramel et al., 2012a). The enhanced formation of ^1^O_2_ in *ch1* was confirmed by measuring ^1^O_2_ in leaves using ^1^O_2_-specific *flu* probes (Dall’Osto et al., 2010; Ramel et al., 2013a) and lipid oxidation markers (Triantaphylidès et al., 2008), or in thylakoids using spin probes and EPR spectroscopy (Ramel et al., 2013a). Moreover, a good correlation was found between the transcriptomic profiles of the *ch1* and *flu* mutants, supporting the fact that a ^1^O_2_ signaling pathway is activated in the *ch1* leaves under high light stress. However, important differences were observed between the two mutants. First, cell death in *ch1* is not dependent on the EXECUTER proteins (Ramel et al., 2013a). Second, the increased production of ^1^O_2_ in the *ch1* mutant occurs in the main natural production site, the PSII reaction center (Krieger-Liszkay et al., 2008; Telfer, 2014), while in *flu* ^1^O_2_ is produced from the membrane-bound chlorophyll precursor Pchlide in the absence of PSII over-excitation (op den Camp et al., 2003; Przybyla et al., 2008). In addition, the *flu* mutant must be grown in continuous light to avoid accumulation of Pchlide, while the *ch1* mutant does not require any particular light regime.

## ^1^O_2_-TRIGGERED ACCLIMATION IN *ch1*

By playing with the light conditions, it was found that *ch1* plants are able to acclimate to ^1^O_2_. Pre-exposure of *ch1* plants to a moderately elevated PFD, which induces a moderate and controlled production of ^1^O_2_, rendered the plants resistant to high light stress conditions that induced lipid peroxidation, loss of chlorophyll and PSII inhibition in non-pretreated *ch1* plants (Ramel et al., 2013a). This acclimation phenomenon was not associated with a reduction in the formation of ^1^O_2_ in the PSII centers, indicating a true acclimation phenomenon. Gene responses to ^1^O_2_ that do not lead to cell death were previously reported in the *lut2 npq1* double mutant of *Arabidopsis* deficient in two ^1^O_2_ quenchers (Alboresi et al., 2011). In this mutant ^1^O_2_ formation was associated with the induction of genes whose function is to protect chloroplasts against the damaging effect of ROS. A ^1^O_2_-mediated stress acclimation phenomenon has also been reported more recently in wild-type *Arabidopsis* (Kim and Apel, 2013; Zhang et al., 2014). In addition, *Arabidopsis* cell suspensions exposed to high light display gene responses that very much resemble the gene expression profile induced in the *flu* mutant by ^1^O_2_, but again this occurs in the absence of cell death (González-Pérez et al., 2011).

Acclimation and increased resistance to photooxidative damage in *ch1* were accompanied by a strong downregulation of the JA pathway (Ramel et al., 2013a; **Figure 2**); almost all genes of the JA pathway were downregulated during the acclimation treatment. Moreover, mutational suppression of JA synthesis led to constitutive phototolerance in *Arabidopsis* leaves: a double mutant generated by crossing the *ch1* mutant with the jasmonate-deficient mutant *dde2* was found to be tolerant to light stress conditions that induced severe photooxidative damage in the *ch1* single mutant (Ramel et al., 2013b). Conversely, photodamage in non-acclimated plants was correlated with JA accumulation. Importantly, exogenous applications of methyl-JA were observed to cancel the acclimation response. These results allow us to refine our understanding of the interaction between the ^1^O_2_ signaling and JA pathways that was revealed earlier in the *flu* mutant. JA appears to act as a ‘decision maker’ between cell death and acclimation, with high concentrations of this phytohormone favoring the onset of cell death (Ramel et al., 2013b). In contrast, the strong inhibition of JA biosynthesis, as observed in *ch1* leaves during acclimation to ^1^O_2_, prevents cell death and allows the initiation of cellular defense mechanisms (**Figure 2**). In other words, JA appears to function as a cell death-promoting phytohormone in the response of plants to high light stress. This is consistent with numerous molecular genetic studies that have provided evidence for the involvement of JAs in the regulation of cell death program under various environmental stress conditions (Reymond and Farmer, 1998; Balbi and Devoto, 2008; Reinbothe et al., 2009; Avanci et al., 2010). Whether other oxylipins such as OPDA and dnOPDA could antagonize the activities of JA during high light stress and/or acclimation in *ch1* remains elusive since the concentration of OPDA did not seem to be affected by the treatments (Ramel et al., 2013a).

The role of JAs in plant cell death regulation involves interactions with other signal molecules such as SA and ET (Reymond and Farmer, 1998; Reinbothe et al., 2009; Ballaré, 2011). The close correlation between JA levels and sensitivity to ^1^O_2_ was not observed for SA, which exhibited little change during photooxidative stress in *ch1* mutant plants, unlike in the *flu* mutant (Ramel et al., 2013a). On the other hand and like in *flu*, microarray-based transcriptomic analyses of *ch1* leaves revealed the induction of a number of genes related to ET such as *ACS2*, *ETR1*, *ERF1*, *ERF5,* and *ERF2*, in response to increased PFDs (Ramel et al., 2013a). Interestingly, the ET-responsive element binding protein ERF72 (also known as AtEBP and RAP2.3) and the ET-inducible protein of unknown function encoded by the *At2g38210* gene were found to be specifically induced during acclimation of the *ch1* mutant to ^1^O_2_ stress. The ERF72 protein is involved in the ET signaling pathway, functioning as a transcription activator that regulates the expression levels of plant defense genes and confers resistance to Bax- and abiotic stress-induced plant cell death (Ogawa et al., 2007). Based on these observations, it is tempting to propose a role for ERF72 in the acclimation mechanism as a cell death suppressor. This attractive possibility is currently investigated in our laboratory using an ERF72-deficient mutant of *Arabidopsis*.

## SECONDARY MESSENGERS OF ^1^O_2_-TRIGGERED ACCLIMATION

Recently, ^1^O_2_ oxidation products of β-carotene, such as β-cyclocitral and dihydroactinidiolide, were found to accumulate in *Arabidopsis* leaves under high light stress (Ramel et al., 2012b; Shumbe et al., 2014). Those carotenoid derivatives were able to induce changes in the expression of ^1^O_2_-responsive genes and to increase plant phototolerance. Since β-carotene is located in the PSII reaction center, its oxidized derivatives β-cyclocitral and dihydroactinidiolide can be considered as upstream messengers in the ^1^O_2_ signaling pathway. Although the exact mode of action of those signaling compounds is not yet known, it has been suggested that they act as reactive electrophile species (RES; Havaux, 2014). Interestingly, acclimation to ^1^O_2_ was also reported in the microalga *Chlamydomonas* following exposure to low, sublethal concentrations of ^1^O_2_ (Ledford et al., 2007). This acclimation process is accompanied by gene transcription changes, with the promoter of many genes containing a palindromic sequence element functioning as an electrophile response element (Fischer et al., 2012). Taken together, these results strongly support the involvement of lipid and/or carotenoid RES in the ^1^O_2_ signaling pathway leading to acclimation to ^1^O_2_ toxicity in photosynthetic organisms. Their mechanisms of action remain to be elucidated and constitute a major research challenge for the future. Also, the possible interactions between carotenoid RES and other metabolite retrograde signals from the chloroplasts (Estavillo et al., 2012) need to be assessed.

## ^1^O_2_ AND CELL DEATH IN WILD-TYPE *Arabidopsis* AND IN OTHER PHOTOSYNTHETIC MODELS

Data on ^1^O_2_-induced cell death in wild-type *Arabidopsis* and in other plant species are scarce. Compared to the *ch1* and *flu* mutants, the responses to high light stress are more complex in the wild-type since several ROS can be produced simultaneously in the chloroplasts and several superimposing signaling pathways can be expected. In particular, it has been proposed that the ^1^O_2_- and EXECUTER-dependent pathway operates in wild-type *Arabidopsis* leaves but, depending on the severity of light stress, it can be superimposed by ^1^O_2_-mediated signaling that does not depend on EXECUTER and is associated with photooxidative damage (Kim and Apel, 2013). Nevertheless, in cultured *Arabidopsis* cells, ^1^O_2_ was reported to be the main ROS produced in high light (González-Pérez et al., 2011). Using ^1^O_2_-specific lipid oxidation markers, Triantaphylidès et al. (2008) found that photooxidative damage to *Arabidopsis* leaves is always associated with ^1^O_2_-induced lipid peroxidation, indicating a central role played by this ROS in the execution of high light-induced cell death.

Mor et al. (2014) have recently shown that transcriptomes of multiple stresses, whether from light or dark treatments, are correlated with the transcriptome of the *flu* mutant. They also detected ^1^O_2_ production in roots in the dark, suggesting that the role of ^1^O_2_ in plant stress regulation and response is more ubiquitous than previously thought and is not restricted to high light. Lipid peroxidation and reactions between superoxide and hydrogen peroxide have been suggested as possible sources of ^1^O_2_ in the dark (Khan and Kasha, 1994; Miyamoto et al., 2007). ^1^O_2_ production can also occur under biotic stress conditions, as indicated by the accumulation of ^1^O_2_-specific lipid oxidation products in leaves during pathogen attacks (Vellosillo et al., 2010; Zoeller et al., 2012). Chlorophyll catabolites have been involved in the photosensitization of the hypersensitive response elicited by *Pseudomonas syringae* in *Arabidopsis* (Mur et al., 2010). Actually, any stress conditions that perturb the assembly of the chlorophyll binding proteins can release uncoupled chlorophyll molecules that can act as ^1^O_2_-producing photosensitizers (Santabarbara and Jennings, 2005). It is then clear that ^1^O_2_ signaling can operate under a variety of environmental conditions and can lead, likely through interactions with other signaling pathways, to physiological responses associated with cell death or acclimation.

Adaptative responses to ^1^O_2_ have also been reported in photosynthetic microorganisms. In the facultative photosynthetic α-proteobacteria *Rhodobacter sphaeroides*, bacteriochlorophylls can act as cellular photosensitizers. A ^1^O_2_ response mechanism was shown to occur in this organism, which involves the alternative sigma factor RpoE (Anthony et al., 2005): when cells are exposed to ^1^O_2_, the complex that RpoE forms with its anti-sigma factor ChrR is disrupted, allowing the binding of RpoE to RNA polymerase and resulting in the activation of target gene expression (Nuss et al., 2013). This transcriptional response was associated with an increased tolerance of Rhodobacter cells to ^1^O_2_. Acclimation to ^1^O_2_ was also reported in the microalga *C. reinhardtii*, with low sublethal concentrations of ^1^O_2_ inducing an increased resistance to higher ^1^O_2_ concentrations (Ledford et al., 2007). Recently, two mediators of ^1^O_2_-dependent gene expression have been identified in this organism: a small zinc finger protein named MBS (Shao et al., 2013) and a cytosolic phosphoprotein, SAK1 (Wakao et al., 2014). SAK1 has been proposed to be a key regulator of acclimation to ^1^O_2_ in *C. reinhardtii* because the SAK1 protein is phosphorylated upon exposure to ^1^O_2_ and the *sak1* knock-out lacks a ^1^O_2_ acclimation response. However, SAK1 homologs have been identified only in chlorophytes. In contrast, the identification of a homologous MBS gene in *Arabidopsis*, and the characterization of mutant and overexpressing lines, revealed a critical and evolutionarily conserved role for the MBS protein in ^1^O_2_ signaling and photooxidative stress tolerance.

## CONCLUDING REMARKS AND OUTLOOK

The study of the two ^1^O_2_-overproducing mutants, *flu* and *ch1*, has greatly improved our understanding of the cellular function of ^1^O_2_ in the model plant *A. thaliana*. Besides its cytotoxicity at high concentrations, ^1^O_2_ may trigger signaling pathways that lead either to acclimation or regulated cell death. Key cell death-promoting and -inhibitory signals have been identified that coexist and counteract one another and which may explain, in agreement with the competition model of cell death initiation (Galluzzi et al., 2015), how an adaptive stress response can promote cell death when unsuccessful (**Figure 2**). Specifically, jasmonate, and possibly other oxylipins, seem to play a central role in the decision between acclimation and cell death in the response to ^1^O_2_ (Danon et al., 2005; Ramel et al., 2013b).

The gene expression responses to ^1^O_2_ in the *flu* and *ch1* mutants show many similarities, as expected for two ^1^O_2_ producers. Nevertheless, important differences were also observed. In particular, the dependence of the response to ^1^O_2_ on the EXECUTER proteins, which was revealed in the *flu* mutant, was not observed in the *ch1* mutant. Furthermore, the *ch1* mutant exposed to high light stress did not exhibit increased expression of *EDS1* encoding the ENHANCED DISEASE SUSCEPTIBILITY1 protein that is required for the modulation of the ^1^O_2_-mediated cell death response in *flu* (Ochsenbein et al., 2006). Similarly, in *Arabidopsis* cell suspensions, EDS1 was not up-regulated during ^1^O_2_-induced cell death (Gutiérrez et al., 2014). These differences suggest that the site of ^1^O_2_ production (thylakoid membranes vs. PSII reaction centers) can modulate the gene expression response. Alternatively, or additionally, the ^1^O_2_-production rate and time might contribute to the differences observed between *flu* and *ch1*. In the *flu* mutant, ^1^O_2_ concentration rises immediately after the dark-to-light shift (within minutes, most likely less) and probably quite transiently since it depends on the photosensitizer Pchlide that has accumulated during the preceding dark period. Conversely, the ^1^O_2_ concentration is likely to rise more gradually in the *ch1* mutant under high light stress, but the production lasts as long as the stress is maintained and the PSII reaction centers are not severely damaged. The lack of a precise and continuous determination of ^1^O_2_ concentrations emphasizes the need for the further development of highly sensitive and quantitative ^1^O_2_ detection methods that will allow the accurate detection of ^1^O_2_ *in planta*, and within cellular compartments (Fischer et al., 2013).

The ^1^O_2_ signaling pathways have been shown to operate in the wild-type but the situation is probably more complex than in the *flu* and *ch1* mutants, with the interaction and the convergence of different signaling pathways (Kim and Apel, 2013). In addition, several lines of evidence indicate that the role of ^1^O_2_ in plant stress response is probably more ubiquitous than usually thought and is not exclusive to excess light energy. Indeed, not only ^1^O_2_ appears to be involved in the response to pathogen attacks, but it can also emanate from compartments other than the chloroplasts and in a light-independent manner. Therefore, a better knowledge of the plant responses to ^1^O_2_ could have important implications not only for the understanding of how plants can adapt to changing and unfavorable climatic environments, but also for the development of plants tolerant to various types of stresses, including biotic stresses.

## Conflict of Interest Statement

The authors declare that the research was conducted in the absence of any commercial or financial relationships that could be construed as a potential conflict of interest.
